# Factors influencing the performance of community health volunteers working within urban informal settlements in low- and middle-income countries: a qualitative meta-synthesis review

**DOI:** 10.1186/s12960-021-00691-z

**Published:** 2021-11-27

**Authors:** Michael Ogutu, Kui Muraya, David Mockler, Catherine Darker

**Affiliations:** 1 Health Systems & Research Ethics Department, KEMRI-Wellcome Trust Research Programme, P.O Box 43640-00100, Nairobi, Kenya; 2 Public Health & Primary Care, Institute of Population Health, School of Medicine, Trinity College Dublin, Dublin, Ireland; 3 Trinity Centre for Global Health, School of Medicine, Trinity College Dublin, Dublin, Ireland; 4 School of Medicine, Trinity College Dublin, Dublin, Ireland

**Keywords:** Community health volunteer, Low- and middle-income countries, Urban, Informal settlement, Performance

## Abstract

**Background:**

There is limited information on community health volunteer (CHV) programmes in urban informal settlements in low- and middle-income countries (LMICs). This is despite such settings accounting for a high burden of disease. Many factors intersect to influence the performance of CHVs working in urban informal settlements in LMICs. This review was conducted to identify both the programme level and contextual factors influencing performance of CHVs working in urban informal settlements in LMICs.

**Methods:**

Four databases were searched for qualitative and mixed method studies focusing on CHVs working in urban and peri-urban informal settlements in LMICs. We focused on CHV programme outcome measures at CHV individual level. A total of 13 studies met the inclusion criteria and were double read to extract relevant data. Thematic coding was conducted, and data synthesized across ten categories of both programme and contextual factors influencing CHV performance. Quality was assessed using both the Critical Appraisal Skills Programme (CASP) and the Mixed Methods Assessment Tool (MMAST); and certainty of evidence evaluated using the Confidence in the Evidence from Reviews of Qualitative research (CERQual) approach.

**Results:**

Key programme-level factors reported to enhance CHV performance in urban informal settlements in LMICs included both financial and non-financial incentives, training, the availability of supplies and resources, health system linkage, family support, and supportive supervision. At the broad contextual level, factors found to negatively influence the performance of CHVs included insecurity in terms of personal safety and the demand for financial and material support by households within the community. These factors interacted to shape CHV performance and impacted on implementation of CHV programmes in urban informal settlements.

**Conclusion:**

This review identified the influence of both programme-level and contextual factors on CHVs working in both urban and peri-urban informal settlements in LMICs. The findings suggest that programmes working in such settings should consider adequate remuneration for CHVs, integrated and holistic training, adequate supplies and resources, adequate health system linkages, family support and supportive supervision. In addition, programmes should also consider CHV personal safety issues and the community expectations.

**Supplementary Information:**

The online version contains supplementary material available at 10.1186/s12960-021-00691-z.

## Background

Populations living in urban informal settlements are often characterized by rapid population growth [1], a high burden of disease [2, 3] and limited access to healthcare [4–6]. To increase healthcare access to such underserved populations and with renewed calls for universal health coverage (UHC), governments have rolled out various community health programmes using Community Health Volunteers (CHVs) as a link between the formal healthcare system and the community [7]. This cadre of workers are referred to using differing terms depending on context such as Community Health Workers (CHWs), Accredited Social Health Activists (ASHAs), CHVs, lay health workers, etc., but for this paper we will be using the term CHV to broadly refer to them. In this study, a CHV is one who is involved in health service delivery with limited training necessary for the implementation of the different primary healthcare interventions [7].

In some countries such as Bangladesh and India [8, 9], CHVs are paid while in others such as Kenya they are not [9]. Studies show that the success of such programmes depends on programme design factors (features of the programme or intervention such as renumeration, recruitment, supervision, workload); health system factors (such as health system financing and governance) and broad contextual factors (for instance gender roles, norms, cultural practices and legislation) surrounding the workings of such programmes [10–12].

Several reviews have examined factors that influence the performance of CHVs in LMICs [7, 12–16]. Nonetheless, the majority of these have focused on rural areas [17]; with less attention paid to CHVs working in urban or peri-urban informal settlements [18]. Literature shows that context matters when it comes to the performance of CHVs. In particular, contextual factors related to the community, economy, environment and health system policy and practice can interact to shape CHV performance [19]. It is, therefore, important to have specific understanding of factors influencing CHV performance in urban contexts. In this study, an urban area was defined as, “a city or town with a high population density and built up features compared to the surrounding areas” (p. 3) [20]. A peri-urban area was defined as, “an area that borders an urban area, between the suburbs and the countryside” (p. 3) [20]. Whereas an informal settlement was defined as, “a residential area where the inhabitants had no security of tenure for their lands or dwellings”, lacked “basic services and city infrastructure, and their housing did not comply with the planning and building regulations” in addition to “being situated in geographically and environmentally sensitive locations” (p. 30) [20].

The current review explored the implementation dynamics including the challenges and successes of CHV-programmes within urban informal settlements, with a focus on CHV performance at individual level. Measurable elements at this level included the behavioural, affective, and cognitive changes in the individual CHV such as self-esteem, motivation, attitudes, competencies, job satisfaction and adherence with standards and procedures [10].

The objective of this study was therefore to identify programme design and contextual factors (broader and health system factors) that influence the performance of CHVs working within urban informal settlements in LMICs. The study adopted a qualitative approach for the review since qualitative approaches are well-suited in analysing human experiences, cultural and social phenomena [21]. Purely qualitative studies plus mixed methods studies that used qualitative methods for data collection and analysis were included.

## Methodology

This systematic review adapted a qualitative meta-synthesis type of review using meta-aggregation technique of data synthesis and reporting [22].

### Search strategy

A search strategy adopted from Kok et al. [10], and modified to focus on qualitative studies in urban and peri-urban informal settlements (Additional file 1) was used for the review, with the assistance of a medical librarian (DM). The Population, Exposure, Outcome and Study (PEOS) framework [21, 23] (Table 1) was used in identifying keywords in the review question. Once the keywords were ascertained, a table listing all the CHV synonyms was adapted from The World Health Organization guideline on health policy and systems support to optimize community health worker programmes [24] and Kok et al. [10] as outlined in Table 2, to guide the search (Additional file 2). This table formed the basis of the search strategy.

**Table 1:** Research questions and search tool used to address the research question

Broad research question	Specific research questions
What factors influence the performance of *CHV*s working within *urban informal settlement*s in *low-and middle-income countries*?	1. Which programme design factors influence individual-level performance of CHVs working within urban informal settlements in low-and middle-income countries?
	2. Which contextual factors (both the broader and health system factors) influence individual-level performance of CHVs working within urban informal settlements in low-and middle-income countries?
*P*opulation	*CHV*s working within *urban informal settlement*s in *low-and middle-income countries*
*E*xposure	CHV programme
*O*utcome	Programme design, health system and broad contextual factors that influence CHV performance at individual level
*S*tudy	Qualitative

**Table 2:** CHV search terms

Community health workers	Village health volunteers	Mother coordinator	Female community health volunteers
Community health nursing	Close-to-community providers	Outreach educators	Health agents
Health auxiliary	Community-based practitioners	Promotora	Health assistants
Frontline health workers	Lady health workers	Shastho Shebika	Health surveillance assistants
Midwives	Barefoot Doctors	Shastho Karmis	
Birth attendant	Community practitioners	Shevika	Lead mother
Outreach workers	Promotoras De Salud	Village health helper	Monitora
Lay health workers	Agentes De Saúde	Village drug-kit manager	Malaria agents
Promotoras	Rural health auxiliaries	Accompagnateur	Health extension workers
Village health workers	Activista	Accredited social health activist	Maternal and child health workers
Volunteer health workers	Agente Comunitario De Salud	Animator	Community health extension workers
Volunteer health workers	Agente Comunitário De Saúde	Asha	Mobile clinic teams
Community health agents	Anganwadi	Auxiliary nurse	
Health promoters	Animatrice	Auxiliary nurse-midwife	Nutrition agents
Community health workers	Barangay health workers	Bridge-to-health team	Nutrition counsellors
Community health aides	Basic health workers	Behvarz	Peer educators
Community health nurses	Brigadista	Care group volunteers	Shasthya Shebika
Community health officers	Colaborador Voluntario	Community case management workers	Socorrista
Community health volunteers	Community drug distributors	Community health care providers	Nutrition counselor
Community health distributors	Community health agents	Community healthcare provider	Community-based volunteer
Community health surveyors	Community health representatives	Community surveillance volunteers	Community health care worker
Community health assistants	Community resource person	Family health workers	

Four health-related databases were used to conduct the search [EMBASE, CINAHL, Web of Science, and Medline (OVID)] between the 8th and 12th of April 2020. Reference lists of all relevant papers were examined for additional citations. To reduce publication bias [23], a search of grey literature was also conducted using Google Scholar. However, no grey literature qualified for inclusion.

**Fig. 1:**
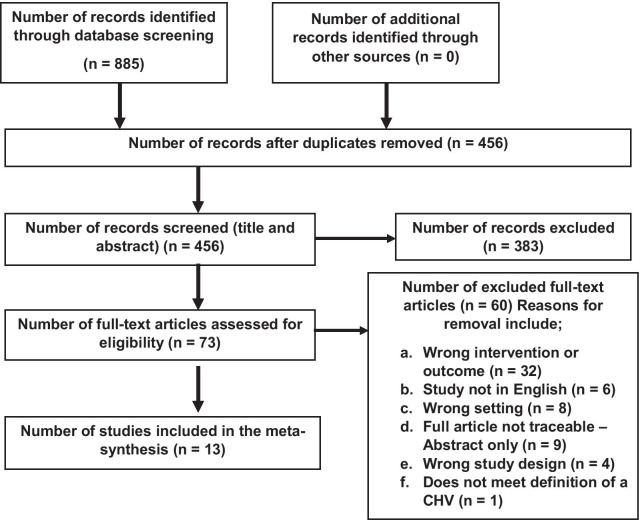
Prisma flow chart adapted from the PRISMA 2009 flow diagram [25]

Covidence systematic review software was used to organize and manage papers which were screened for duplicates and eligible studies identified. The PRISMA flow-chart adapted from the PRISMA 2009 flow diagram [25] (Fig. 1) summarized the search process resulting in 13 studies that met the inclusion criteria and were fully reviewed.

Three reviewers (MO, CD and KM) were involved in this review process [26]. All potential studies were reviewed by MO while CD independently reviewed a random selection of one third of all reviewed studies at each stage. The review process involved a two-stage screening process, with articles being screened based on the title and abstract, followed by a full text review once all reviewers agreed on the results of the first screening. Only those titles and abstracts that met the inclusion criteria as shown in Table 3 were reviewed in full. In the second screening stage, each article was read in full and assessed for inclusion. In case of a disagreement in review at any stage, the third reviewer (KM) was drawn upon to build consensus. No article was reported to have any missing or incomplete information.

**Table 3:** Inclusion criteria

1.	Population	Studies involving CHVs working within urban informal settlements in LMICs
2.	Exposure	Studies describing factors related to a programme design
3.	Outcome	Studies that described factors that influenced CHV performance at the individual level. Measurable elements at individual level include the cognitive, affective, and behavioural changes in the individual CHV such as self-esteem, motivation, competency levels, adherence with standards, job satisfaction levels, etc
4.	Studies	Only studies that used qualitative methods for collection of data and analysis
5.	Setting	Urban and peri-urban informal settlements in low- and middle-income countries
6.	Time frame	None
7.	Language	Only studies published in English
8.	Publication type	All published and unpublished literature

### Assessment of risk of bias

Since this was a synthesis of qualitative studies, no assessment of risk of bias was conducted for the included studies. Instead, a quality assessment for the included studies was conducted as described below [27].

### Assessment of quality

Quality assessment was completed using the Critical Appraisal Skills Programme (CASP) tool for qualitative studies [28]. For mixed methods studies, quality assessment was done using the Mixed Methods Assessment Tool (MMAT) [29]. The quality of included articles was assessed by all three reviewers with MO reviewing all the articles. Any disagreements were resolved through discussion or by involving the third reviewer (CD or KM depending on who was the 2nd reviewer). Since the aim of the review was to obtain a better understanding of the factors that influence the performance of CHVs in urban informal settlements, studies were not excluded based on quality [16, 30]. However, both the CASP and MMAT tools were used as part of the Confidence in the Evidence from Reviews of Qualitative research (CERQual) approach for accessing confidence in the findings of the systematic review [27, 31].

### Assessment of the certainty of review findings

The certainty of the review findings was assessed using the CERQual approach. Confidence levels were assigned based on an overall assessment of the studies’ methodological concerns, relevance, coherence, and adequacy. Studies determined to possess minor methodological issues and high adequacy, coherence and relevance were assigned high CERQual confidence. While those with major methodological limitations and low coherence or relevance assigned low confidence. Studies with mixed scores were assigned moderate confidence [32, 33]. These levels of confidence are defined in Tables 4 and 5.

**Table 4:** CERQual summary of qualitative findings

Review findings				Confidence in the evidence	Explanation of confidence in the evidence assessment	Contributing studies
Programme design factors	Financial incentives					
	Financial incentives were a great motivation for CHVs and those CHVs who depended on their role for financial support are more likely to remain as CHVs and more likely to be active CHVs			Moderate	Moderate concerns regarding methodological limitations	Sarma (2020), Aseyo (2018), George (2017), Osindo (2016), Odhiambo (2016), Swartz (2015), Alam (2012), Boros (2011), Alam (2009), Laston (1993)
Factors related to human resource management	Non-financial factors					
	Job satisfaction and self-identity	Appreciation of the work carried out by members of the community, and being recognized personally because of their home visits, proved to be motivating factors for the community health workers. The role also brought them a sense of identity and satisfaction		Moderate	Serious concerns regarding methodological limitations and moderate volume of data available	George (2017), Osindo (2016), Lopes (2012), Laston (1993)
	Community trust	Enhanced CHW motivation while lack of it led to demotivation				
	Social status, prestige, and respect	Being a volunteer brought prestige, respect and recognition/acknowledgement to the CHVs thus being a source of motivation for them to continue working as CHVs		Moderate	Moderate concerns regarding methodological limitations and data adequacy	George (2017), Osindo (2016), Alam (2012), Lopes (2012), Alam (2009) Laston (1993)
	Job opportunities	Becoming a volunteer increased opportunity of getting a paid job		Moderate	Moderate concerns regarding methodological limitations and serious concerns about data adequacy	Goudet (2018) Swartz (2015), Laston (1993),
	Training					
	Increased knowledge and awareness levels of CHVs thus making them more credible to the community			Moderate	Moderate concerns on methodological limitations and the volume of data	Sarma (2020), Goudet (2018), Aseyo (2018), Osindo (2016), George (2017), Laston (1993)
	Supplies and resources					
	Lack of supplies impacted negatively on community visits to health facilities hence hindering CHV performance			Moderate	Moderate methodological limitations and serious concerns about the low volume of data	Sarma (2020), George (2017), Osindo (2016), Odhiambo (2016), Aseyo (2018), Laston (1993)
	Lack of proper protective equipment by CHVs resulted in low treatment coverage					
	Supplies and materials play not only a functional role in the execution of CHVs' duties, but also a symbolic role in CHV relationships with their communities					
	Provision of job aids help CHVs feel more confident in counselling and giving targeted messages					
	“Receiving some type of an identification badge, a sari, an umbrella or a bag would be helpful in their work.”					
	Health system linkage					
	(Relationship between CHVs and other healthcare workers)		Attitudes of other health staff had an impact on how CHVs feel and performed. Lack of acknowledgement and recognition by the other healthcare staff caused demotivation in the profession	Moderate	Moderate concerns on methodological limitations and the volume of data	George (2017), Osindo (2016), Odhiambo (2016), Lopes (2012), Boros (2011), Laston (1993)
	Family support					
	Relationship between CHVs and their families		Family support of the role played by a CHV is key to the CHV’s level of activity. Disapproval leads to CHV dropout	Moderate	Moderate concerns on methodological limitations and the volume of data	George (2017), Alam (2012), Alam (2009), Laston (1993)
			Gendered Household roles and duties: Female CHVs with no or fewer household responsibilities are more likely to remain as CHVs	Low	Moderate concerns on methodological limitations and the low volume of data	Alam (2009), Laston (1993)
Programme design factors	Supportive supervision					
	Supportive supervision seen as a source of motivation for CHVs			Moderate	Moderate concerns on methodological limitations and serious concerns about the low volume of data	Aseyo (2018), Karuga (2017), Odhiambo (2016)
Factors related to human resource management	CHV personal characteristics					
	Age		Affected interaction between the CHEWs and CHVs. A supervisor’s age in relation to the supervisees affected whether the supervisor would be able to provide adequate supervision	Low	Serious concerns regarding methodological limitations, minor concerns on partial relevance and serious concerns about the low volume of data	Karuga (2017)
	Prior experience with health condition		Prior experience of ill health or condition seen as a motivation of becoming a CHV	Low	Serious concerns on methodological limitations and the low volume of data	George (2017)
Broad contextual factors	Community context factors					
	Insecurity		Insecurity and inaccessibility of certain neighbourhoods impacted negatively on CHV performance	Moderate	Moderate concerns on methodological limitations and the low volume of data	Odhiambo (2016), Osindo (2016), Lopes (2012)
	Gender		Women are seen as the ‘natural’ providers of care and it is assumed that this is what shapes women’s ability and their motivation to engage in CHV work	Low	Serious concerns on methodological limitations and the low volume of data	Swartz (2015)
	African ethic of Ubuntu		It is natural for African people to care for another	Low	Serious concerns on methodological limitations and the low volume of data	Swartz (2015)
	Economic contextual factors					
	Demand for financial or material support		Households expected tangible support from CHVs, which some CHVs provided from their own resources out of sympathy and to increase their acceptance in the community	Moderate	Moderate concerns on methodological limitations and the volume of data	Aseyo (2018), Goudet (2018), Odhiambo (2016), Osindo (2016)

Overall CERQual rating of confidence in the finding, based on four levels of confidence in the evidence contributing to the finding:

• High—it is highly likely that the review finding is a reasonable representation of the phenomenon of interest

• Moderate—it is likely that the review finding is a reasonable representation of the phenomenon of interest

• Low—it is possible that the review finding is a reasonable representation of the phenomenon of interest

• Very low—it is not clear whether the review finding is a reasonable representation of the phenomenon of interest

**Table 5:** Full evidence profile for qualitative findings of factors that influence the performance of CHVs working within urban informal settlements in low- and middle-income countries

Programme design factors	Review finding		Contributing studies	Methodological limitations	Coherence	Relevance	Adequacy	CERQual confidence	Explanation of confidence in the evidence assessment
Factors related to human resource management									
Financial incentives	Financial incentives were a great motivation for CHVs and those CHVs who depended on their role for financial support are more likely to remain as CHVs and more likely to be active CHVs		Sarma (2020), Aseyo (2018), George (2017), Osindo (2016), Odhiambo (2016), Swartz (2015), Alam (2012), Boros (2011)	Moderate methodological limitations (2 studies with serious limitations (unclear recruitment strategies, no information on the researcher’s effect and data analysis not sufficiently rigorous), 6 moderate (No clear statement of the aim of the research, no adequate information on researcher’s effect, no information on non-response and no mention on ethical approval) and 2 with no/minor concerns)	No/very minor concerns about coherence	No/very minor concerns about relevance	No/very minor concerns about adequacy (10 studies that together offered moderately rich data)	Moderate	Moderate concerns regarding methodological limitations
			Alam (2009), Laston (1993)						
Non-financial incentives									
Job satisfaction and self-identity	Appreciation of the work carried out by members of the community, and being recognized personally because of their home visits, proved to be motivating factors for the community health workers. The role also brought them a sense of identity and satisfaction		George (2017), Osindo (2016), Lopes (2012), Laston (1993)	2 serious methodological limitations (George: No adequate information on the recruitment strategy and the researcher’s effect and Lopes: No clear recruitment strategy, no information on the researcher’s effect, no adequate information on data analysis and threats to participants by the community not addressed, i.e. ethical concerns)	No/very minor concerns about coherence	No/very minor concerns about relevance	moderate concerns about adequacy	Moderate	Serious concerns regarding methodological limitations and moderate volume of data available
Community trust	Community trust enhanced CHW motivation while lack of it led to demotivation			Two studies with moderate methodological limitations (Osindo: no information on the researcher’s effect and Laston: No mention of ethical approval)					
Factors related to human resource management									
Social status, prestige and respect	Being a volunteer brought prestige, respect and recognition/acknowledgement to the CHVs thus being a source of motivation for them to continue working as CHVs		George (2017), Osindo (2016), Alam (2012), Lopes (2012), Alam (2009) Laston (1993)	Moderate methodological limitations (2 studies with serious methodological limitations—Lopes: No clear recruitment strategy, no information on the researcher’s effect, no adequate information on data analysis and threats to participants by the community not addressed and George: No clear recruitment strategy, no information researcher’s effect) and 4 studies with no/minor limitations	No/very minor concerns about coherence	No/very minor concerns about relevance	Moderate concerns about adequacy	Moderate	Moderate concerns regarding methodological limitations and data adequacy
Job opportunities	Becoming a volunteer increased opportunity of getting a paid job		Goudet (2018) Swartz (2015), Laston (1993)	Moderate methodological limitations (1 study with serious methodological limitations—Swartz (2015) No adequate information on recruitment strategy, no enough data to support findings and no clear statement of findings)	No/very minor concerns about coherence	No/very minor concerns about relevance	Serious concerns about adequacy	Moderate	Moderate concerns regarding methodological limitations and serious concerns about data adequacy
Training									
Training increased knowledge and awareness levels of CHVs thus making them more credible to the community			Sarma (2020), Goudet (2018), Aseyo (2018), George (2017), Osindo (2016), Laston (1993)	Moderate methodological limitations (2 studies with serious limitations—George: No clear recruitment strategy, no information on the researcher’s effect, and Osindo: no information on the researcher’s effect and 1 study with moderate limitations—Sarma)	No/very minor concerns about coherence	No/very minor concerns about relevance	Moderate concerns about adequacy	Moderate	Moderate concerns on methodological limitations and the volume of data
Factors related to human resource management									
Supplies and resources									
Lack of supplies impacted negatively on community visits to health facilities hence hindering CHV performance			Sarma (2020), George (2017)	Moderate methodological limitations (2 study with serious limitations—George: No clear recruitment strategy, no information researcher’s effect and Lopes: No clear recruitment strategy, no information on the researcher’s effect, no adequate information on data analysis and threats to participants by the community not addressed—and 5 moderate limitations—3 with no adequate information on researcher’s effect, no sampling and recruitment strategies and 2 with no adequate information on researcher’s effect)	No/very minor concerns about coherence	No/very minor concerns about relevance	Moderate concerns about adequacy	Moderate	Moderate methodological limitations and serious concerns about the low volume of data
Lack of proper protective equipment by CHVs resulted in low treatment coverage			Osindo (2016), Odhiambo (2016)						
Supplies and materials play not only a functional role in the execution of CHVs' duties, but also a symbolic role in CHV relationships with their communities			Aseyo (2018)						
Provision of job aids help CHVs feel more confident in counselling and giving targeted messages			Sarma (2020)						
“Receiving some type of an identification badge, a sari, an umbrella or a bag would be helpful in their work.”			Laston (1993)						
Health system linkage									
Relationship between CHVs and other healthcare workers	Attitudes of other health staff had an impact on how CHVs feel and performed. Lack of acknowledgement and recognition by the other healthcare staff caused demotivation in the profession		George (2017), Osindo (2016), Odhiambo (2016), Lopes (2012), Boros (2011), Laston (1993)	Moderate methodological limitations (2 studies with serious limitations—George: no clear recruitment strategy, no information researcher’s effect, Lopes: No clear recruitment strategy, no information on the researcher’s effect, no adequate information on data analysis and threats to participants by the community not addressed—and 4 studies with minor limitations)	No/very minor concerns about coherence	No/very minor concerns about relevance	Moderate concerns about adequacy	Moderate	Moderate concerns on methodological limitations and the volume of data
Factors related to human resource management									
Family support									
Relationship between CHVs and their families	Family support of the role played by a CHV is key to the CHV’s level of activity. Disapproval leads to CHV dropout		George (2017), Alam (2012), Alam (2009), Laston (1993)	Moderate methodological limitations (1 study with serious limitations—no clear recruitment strategy, no information on the researcher’s effect and 3 studies with no/very minor limitations)	No/very minor concerns about coherence	No/very minor concerns about relevance	Moderate concerns about adequacy	Moderate	Moderate concerns on methodological limitations and the volume of data
	Gendered Household roles and duties: Female CHVs with no or fewer household responsibilities are more likely to remain as SS		Alam (2009), Laston (1993)	Moderate methodological limitations (1 study—Laston with minor limitation—no mention of ethical approval)	No/very minor concerns about coherence	No/very minor concerns about relevance	Serious concerns about adequacy	Low	Moderate concerns on methodological limitations and the low volume of data
Supportive supervision									
Supportive supervision seen as a source of motivation for CHVs			Aseyo (2018), Karuga (2017)	Moderate methodological limitations (1 study with moderate and 1 study with serious limitations—no information on sampling, recruitment strategy, No clear research question, potential for respondent bias and no adequate rationale for using a mixed method design to address the research question and 1 study with minor concern on researcher’s effect)	No/very minor concerns about coherence	No/very minor concerns about relevance	Serious concerns about adequacy	Moderate	Moderate concerns on methodological limitations and serious concerns about the low volume of data
			Odhiambo (2016)						
CHV personal characteristics									
Age		Age was a factor that affected interaction between the CHEWs and CHVs. A supervisor’s age in relation to the supervisees affected whether the supervisor would be able to provide adequate supervision	Karuga (2017)	Serious methodological limitations (No clear research question, there was a potential for respondent bias since the implementors of the intervention are the ones who also evaluated it and no adequate rationale for using a mixed method design to address the research question)		Minor concern about relevance—partial relevance (study had rural components)	Serious concerns about adequacy (1 study with thin data)	Low	Serious concerns regarding methodological limitations, minor concerns on partial relevance and serious concerns about the low volume of data
Prior experience with health condition		Prior experience of ill health or condition seen as a motivation of becoming a CHV	George (2017)	Serious methodological limitations (No clear recruitment strategy, no information researcher’s effect)		No/very minor concerns about relevance	Serious concerns about adequacy (1 study with very thin data)	Low	Serious concerns on methodological limitations and the low volume of data
Community contextual factors									
Insecurity		Insecurity and inaccessibility of certain neighborhoods impacted negatively on CHV performance	Odhiambo (2016), Osindo (2016), Lopes (2012)	Moderate methodological limitations (1 study with serious limitations – No clear recruitment strategy, no adequate information on data analysis and researcher’s effect, and threats to participants by the community not addressed - and 2 with moderate limitations – no adequate information on researcher’s effect)	No/very minor concerns about coherence	No/very minor concerns about relevance	Serious concerns about adequacy	Moderate	Moderate Moderate concerns on methodological limitations and the low volume of data.
Gender		Women are seen as the ‘natural’ providers of care and it is assumed that this is what shapes women’s ability and their motivation to engage in CHV work.	Swartz (2015	Serious methodological limitations (No adequate information on recruitment strategy, no enough data to support findings and no clear statement of findings)		No/very minor concerns about relevance	Serious concerns about adequacy (1 study with thin data)	Low	Serious concerns on methodological limitations and the low volume of data.
African ethic of Ubuntu		It is natural for African people to care for another	Swartz (2015	Serious methodological limitations (No adequate information on recruitment strategy, no enough data to support findings and no clear statement of findings)		No/very minor concerns about relevance	Serious concerns about adequacy (1 study with thin data)	Low	Serious concerns on methodological limitations and the low volume of data.
Economic contextual factors									
Demand for financial or material support		Households expected tangible support from CHVs, which some CHVs provided from their own resources out of sympathy and to increase their acceptance in the community.	Aseyo (2018), Goudet (2018), Odhiambo (2016), Osindo (2016),	Moderate methodological limitations (2 studies with moderate limitations – No adequate information on researcher’s effect and no information on sampling and recruitment strategy respectively - and 2 study with minor limitations)	No/very minor concerns about coherence	No/very minor concerns about relevance	Moderate concerns about adequacy	Moderate	Moderate concerns on methodological limitations and the volume of data.

Overall CERQual rating of confidence in the finding, based on four levels of confidence in the evidence contributing to the finding:

∙ High—it is highly likely that the review finding is a reasonable representation of the phenomenon of interest

∙ Moderate—it is likely that the review finding is a reasonable representation of the phenomenon of interest

∙ Low—it is possible that the review finding is a reasonable representation of the phenomenon of interest

∙ Very low—it is not clear whether the review finding is a reasonable representation of the phenomenon of interest

### Data extraction and management

All reviewers were involved in data extraction, with the primary author (MO) reviewing all articles while the other reviewers reviewed an equal number of the same articles. Both first (participant’s words) and second order (author’s interpretation of the participant’s words) constructs were extracted in the review exercise. This allowed the reviewers to examine both raw data as well as the author’s interpretation, thus ensuring the results of the review were based on the original experiences of the participants as well as second order analysis [23, 32]. An adapted data mining tool from Joanna Briggs Institute (JBI) was used for the data extraction process [30, 34]. The extracted data included; the main author’s name, publication year, setting (country of study and setting), intervention type, research methods, results (reported factors that influence CHV performance and related themes) and elements from both the CASP and MMAT tools. The data extraction tool was piloted on three studies before being applied to other studies to test for its validity, and adjustments made accordingly by two of the authors (MO and CD).

### Data synthesis

Thematic framework analysis approach was used for data synthesis [35]. Findings were continuously discussed with the rest of the reviewers to ensure the results reflected original data [23].

## Results

This section begins by presenting an overview of the screening results followed by a description of the findings of the systematic review organized into broad thematic areas (programme design factors and broad contextual factors). The sub-themes identified under the programme design factors included financial and non-financial incentives, training, supplies and resources, health system linkage, family support, supportive supervision, and CHV characteristics. While the sub-themes identified under the broad contextual factors included both the community and economic contextual factors. Each of these are discussed in turn below.

### Results of screening

The search resulted in 885 articles, 13 of which [9, 36–47] met the inclusion criteria (see PRISMA flowchart in Fig. 1).

### Study characteristics

Most of the articles [9, 36, 37, 39–47] were published from the year 2009 onwards, with only one article [38] published in 1993. Of the articles, seven [36, 38, 41, 42, 44–46] were qualitative and six [9, 37, 39, 40, 43, 47] were mixed method studies. The review covered five countries in total with seven [39, 41–45, 47] of the studies undertaken in Sub-Saharan Africa (Kenya and South Africa), five [9, 36–38, 40] in Asia (Bangladesh and India) and one [46] from Latin America (Brazil). The papers covered a wide range of health issues. Of the programmes which specified health issues, the largest number (six) focused on maternal, neonatal and child health [9, 36–40]. Other health issues of focus included HIV/AIDS, tuberculosis and nutrition [41], schistosomiasis [42] and hygiene-related behaviour change [43] (Table 6).

**Table 6:** General characteristics of studies included in the review

Category	Sub-category	No.	Study references
Year of publication	1993	1	[39]
	2009	1	[9]
	2011	1	[45]
	2012	2	[36, 41]
	2015	1	[42]
	2016	2	[35, 43]
	2017	1	[34]
	2018	2	[40, 44]
	2019	1	[37]
	2020	1	[38]
Publication type	Qualitative	7	[34–36, 39, 42, 43, 45]
	Mixed method	6	[9, 37, 38, 40, 41, 44]
Countries of origin	Kenya	5	[35, 37, 40, 43, 44]
	South Africa	2	[42, 45]
	Bangladesh	4	[9, 38, 39, 41]
	India	1	[34]
	Brazil	1	[36]
Health issues addressed	Maternal, neonatal and child health	6	[9, 34, 38–41]
	HIV/AIDS, tuberculosis, and nutrition	1	[42]
	Schistosomiasis	1	[43]
	Hygiene-related behaviour change	1	[44]
	Not specified	4	[35–37, 45]

#### Programme design factors

##### Financial incentives

Financial incentives were identified in ten studies as indicated in Tables 4 and 5 as one of the key motivating factors for CHVs. The studies were from Kenya [42, 43, 45], South Africa [41, 44], India [36] and Bangladesh [9, 37, 38, 40]. Those CHVs who depended on their role for financial support were more likely to remain as CHVs and more likely to be active [9, 36–38, 40–45] compared to their counterparts. In these studies, CHVs mainly depended on the financial incentives to sustain their livelihoods. As such, they easily dropped out of programmes that offered no financial incentives in preference for personal income generating activities, as they found it difficult to provide for their families including paying for their children’s education [9, 40, 41, 43–45]. To help compensate for the lack of financial income, CHVs undertook supplemental income generating activities such as selling cooked food/’street food’ in their local area or undertaking casual paid domestic work [43, 45]. This consequently, led to limited time devoted to CHV work [43]. The CHVs also partnered with local non-governmental organizations (NGOs) as a way of earning an income [43].

##### Non-financial incentives

Tables 4 and 5 describe the non-financial incentives identified in the reviewed studies and their corresponding CERQual confidence levels. These were noted to either impact positively or negatively on the CHVs motivation and their performance in turn. For instance, in Kenya, India, Bangladesh and Brazil, being appreciated and recognized by the community for the role they played, gave the CHVs a sense of identity [36, 38, 45, 46]. The CHVs also felt that they were saving lives and improving the health of their community members, which motivated them to continue playing their role [36, 38, 46]. Conversely, lack of acknowledgement by both the community and health workers in the formal healthcare system was reported to be a demotivating factor for CHVs [36, 45, 46].

In India, the trust earned from the community was regarded by ASHAs, as one of the greatest incentives for continuation in their role. The CHVs were also held in high regard because of this trust. The CHVs were considered a valuable source of both health-related and other information in the community, which made them feel appreciated, and motivated them to continue in their role [36].

In India, Bangladesh, Brazil, and Kenya social status, prestige and respect were reported as important facilitating factors for CHVs working in urban informal settlements [9, 36, 38, 40, 45, 46]. At the same time in India, South Africa and Kenya some CHVs commenced in the role with the hope of eventually gaining salaried employment with NGOs or clinics [38, 39, 41].

##### Training

Seven studies reported that CHVs received some training to help in carrying out their roles and duties [9, 39, 40, 42–44, 47]. Of these, six reported an association between training and CHV performance. In this six studies, training was reported to have increased the CHVs’ knowledge level of different aspects of their work such as team supervision and working with the community; hence making them more credible to the community [36–39, 47]. Training also increased CHVs’ chances of getting employment [39]. However, in one of the studies, the CHVs reported different topic areas, lengths and format of trainings; that left CHVs with knowledge gaps on technical aspects of their work such as community mobilization, behaviour change techniques, risk factors identification and symptom recognition [43].

##### Supplies and resources

The availability (or lack thereof) of supplies and resource also emerged as another dominant theme in this review. A total of six studies reported on the importance of availability of supplies and resources, and their influence on CHV performance [36–38, 42, 43, 45] as indicated in Tables 4 and 5. Health system constraints were reported to negatively impact on the CHVs’ performance. This was because the CHVs (who work on the demand side of the health system where they motivate and accompany the community for services) felt that their efforts to link the community with the health facilities was not appreciated or valued. As part of their role, CHVs made effort to either bring or refer patients/community members to facilities—only to find that the services they referred the patients to were unavailable, e.g. due to resource stockouts. This in turn made them lose their credibility in the eyes of their community members [36].

In Kenya, the lack of supplies such as gloves, gumboots and first aid kits constrained the CHVs’ ability to deliver healthcare at the household/community level as it exposed them to health risks [42, 43, 45]. In addition, CHVs were reported to attract more positive attention from households when they wore clothes with writing or logos associated with the government or NGOs operating in the community. As such, the lack of supplies such as clothing and badges for identification in the community demotivated CHVs and impacted on their ability to optimally perform their duties [38, 42, 43]. A study in Bangladesh recommended the use of job aids (specifically counselling cards on what to do so that the CHVs pass consistent messages) for CHVs, to help them feel more confident in carrying out their roles and duties [37].

##### Health system linkage

Six studies (see Tables 4 and 5) reported on the working relationship between the CHVs and the formal healthcare staff [36, 38, 42, 44–46]. Poor attitudes towards CHVs by formal healthcare staff were reported to negatively impact on how CHVs felt about their role and how they performed. For example, in India and Brazil, CHVs felt that healthcare workers in the formal system did not value them or the work they did [46]. This was primarily experienced in the referral hospitals [36]. In South Africa, lay health workers felt that nurses did not respect them and vice versa [44]. In all these studies, more positive and improved communication was cited as a potential solution to the problem of challenging relationships between CHVs and formal healthcare workers.

##### Family support

Family support of the role played by the CHV was reported to be key to the CHV’s level of activity, and their motivation and ability to continue in the role [9, 36, 38, 40]. Conversely, disapproval particularly by family, of the CHV role (e.g. as a result of gendered norms related to women being outside of the home late at night, unaccompanied by male relatives or of women working outside the home in general) often led to dropouts [9, 38]. In particular for married women, both the husband and the mother-in-law were reported to be the most important people to the success of CHVs as a result of family hierarchies and related seniority [36, 38]. Their level of moral support and assistance with household chores was significant to the success of CHV work in terms of retention. In Bangladesh for example, gendered household roles was reported to negatively impact on CHVs’ performance in that those with more work load at home were more likely to drop out of CHV programmes [9, 38].

##### Supportive supervision

Three studies [42, 43, 47], all conducted in Kenya reported supportive supervision of CHVs as a factor that positively influenced their performance in urban and peri-urban informal settlements. In all these studies, although a supportive supervisor (community health extension worker—CHEW) was unable to change the less-than-optimal work environments and systems, they played an instrumental role in assisting the CHVs identify and manage complex cases. This support by CHEWs was noted to be an important source of motivation for the CHVs in the Kenyan studies.

##### CHV personal characteristics

Personal characteristics reported to influence the performance of CHVs working in urban informal settlements included age [47] and prior experience with a health condition [36]. In Kenya, age was reported to negatively impact on the interaction between the CHVs working in Nairobi’s informal settlements and their supervisors (CHEWs) [47]. CHEWs who were younger in age found it difficult to supervise CHVs who were older than them; whereas in India, a CHV’s prior personal experience with ill health or a specific health condition was reported to positively impact on CHV performance [36]. For example, those CHVs who had negative experiences as young mothers or lacked the appropriate knowledge on how to care for themselves or their new-borns post-delivery, felt an ‘obligation’ to assist other women so that they did not have similar experiences. This desire to help was a motivating factor to enrol as a CHV.

#### Broad community contextual factors

At the broader contextual level, two main factors as indicated in Tables 4 and 5 were identified from the review as impacting on CHV performance in the community. These were personal safety issues and economic contextual factors. Other factors reported to influence CHV performance included gender and the African culture of Ubuntu.

##### Personal safety issues

Perceived lack of personal safety (insecurity) was reported in three studies in Kenya and Brazil to negatively impact on CHVs’ motivation to work in certain neighbourhoods within urban informal settlements. This in turn adversely impacted on their performance [42, 45, 46]. Some of the reasons cited for insecurity in particular neighbourhoods included high rates of criminal activities such as drug dealing and use, prostitution and intra-family violence occurring within local communities [46].

##### Economic contextual factors

In Kenya, economic factors specifically the demand from community members for financial or material support from CHVs, impacted on their performance and role continuity. [39, 42, 43, 45, 48]. Sometimes, the CHVs gave in to these demands out of sympathy and to increase their acceptance in the community. Other times, the CHVs lost favour when they were unable to provide the requested support in the visited households. Similarly, in other occasions they were completely rejected by the households leading to CHV dropouts [43].

##### Other broader contextual factors

Other broader contextual factors reported to influence CHV performance at the community level especially is South Africa were gender and the African culture of caring or *ubuntu*—a philosophy of shared collective humanness and responsibility [41]. In South Africa women were perceived as the natural providers of care, thus gender was reported to positively shape their ability and drive to engage in CHV-related work [41]. The South Africans also practised a culture of caring or *ubuntu* which was noted to be a motivating factor especially amongst the older CHVs. It is these CHVs who were seen to have the real passion for caring for people and were not driven by economic motivation. Of note was that this culture was considered as being ‘under threat’ in recent times because of contemporary urban lifestyles; and since the younger CHVs were deemed as only being interested in employment and personal career development [41].

## Discussion

This is one of the few reviews that have documented factors that influence the individual-level performance of CHVs working within urban and peri-urban informal settlements in LMICs. Key factors found to influence the performance of CHVs working in these settings included: financial and non-financial incentives; training; the availability of supplies and resources; relationship between the CHVs and other healthcare workers; relationship between CHVs and their families; availability of supportive supervision; personal safety issues and demand for material and economic support by households at the community level. All these factors have been reported amongst CHVs working in the rural setting [17] except those to do with the broader context. These include insecurity (personal safety of CHVs) and the demand for material and economic support by households, which were reported in this review to be predominant amongst CHVs working in urban and peri urban informal settlements.

From the review, CHVs working in urban informal settlements mainly depended on CHV work to financially care for their families [9, 36, 40, 41, 43, 44]. As a result of this dependence, CHVs dropped out of programmes with no financial incentives in preference of personal income generating activities [43]. This finding supports previous reviews by Bhutta et al., (2010) [14], Lehmann & Sanders (2007) [7] and Kok et al., (2014) [10]. According to Bhutta et al., and Lehmann & Sanders’ reviews both conducted in LMIC settings, CHVs were mostly poor people living in low-income communities; and as such could not overlook opportunities to earn an income for voluntary work. Consequently, they were easily demotivated when a programme did not offer financial incentives and dropped out of CHV programmes. To control for such attrition, Bhutta et al. recommended regular, performance based financing incentives or hiring CHVs as full-time waged employees, rather than as part time volunteers [14]. Nonetheless, given the significant resource constraints and system challenges experienced in LMICs, sustainability of such monetary incentives are bound to face challenges due to insufficient funding or irregular payments [7]. Furthermore, evidence from literature suggests that approaches like performance based incentives sometimes resulted in neglect of unpaid tasks [10], or over-reporting of tasks by CHVs [49]. Therefore, for such motivational incentives to work, there is a need for a mix of both financial and non-financial incentives taking context into account, given the high cost of living in the urban settings.

Non-monetary incentives such as community trust, respect, appreciation, and acknowledgment were noted to be important to the success of CHV programmes. In this review, they brought a sense of job satisfaction, respect, self-identity and status to the CHVs [9, 36, 38, 40, 46]. This finding mirrors that of Glenton et al., (2013) [27]. In their review, CHVs especially those from LMICs were motivated by the respect and social status that they received from their own homes and communities. The social recognition accorded to the CHVs was because of their training, moral standing as ‘good-hearted volunteers’ and social importance. This implies that CHV programmes can build on these non-financial incentives and intrinsic motivators to strengthen CHV programmes and ensure their success and continuity.

As in this review, Glenton et al., and Sarma et al., also found that some CHVs hoped that the community role would lead to future employment [50] or further education [27]. To maintain these incentives in the community, there is need to strengthen the community’s trust in the CHVs and CHV programmes in general. This can partly be achieved by having good, visible relationships between the CHVs and the formal healthcare system [27]. Such good working relationships include visits from supervisors, visible contact with health professionals, making referrals to health facilities and accompanying programme recipients to referral facilities [51–54].

Training increased CHVs’ knowledge and awareness levels on the different aspects of their roles thus giving them more confidence and credibility to carry out their work in the community. This finding supports previous works by Glenton et al., [16], Jaskiewicz et al.,[15], Bhutta et al.,[14], Kane et al. [13], and Lehmann & Sanders [7]. In these reviews, training complemented by practical skills improved CHVs’ knowledge and performance. Nonetheless, in a Kenyan study, it was found that CHVs working in both urban and peri-urban informal settlements who received training from NGOs focused on task-specific knowledge and as such lacked uniformity in approaches, content and length despite all the CHVs working in the same setting [43]. This difference in training amongst CHVs in the same setting resulted in knowledge gaps for some CHVs, on some of their roles. This could in turn negatively impact on their credibility given the difference in competency levels. Thus adversely impacting on their abilities to deliver on existing tasks [55]. There is, therefore, a need for more integrated training programmes that cover a wide range of tasks, and that draw on existing national CHWs training manuals.

CHVs lost their credibility as a result of low/no supplies in health facilities [36]. This finding mirrors that of Jaskiewicz et al., and Sarma et al., [15, 50]. In the review by Jaskiewicz et al., CHVs lost the respect of the community because of the disruption in hospital supplies, medicines, and equipment. According to both Sarma et al., and Jaskiewicz et al., CHVs need the trust of the community and whenever that trust is compromised, CHVs become ineffective [15, 50]. Additionally, exposure to risks due to lack of personal protective equipment and loss of identity due to lack of branded clothing or badges [42, 43] and job aids [37] also impacted on the CHVs’ level of performance. This finding concurs with studies by Jaskiewicz et al., [15], Sunguya et al., [56] and Scott et al., [17]. In these studies, regular supply of required resources was needed to ensure that CHVs were effective in their roles and were trusted and respected by their respective communities.

Lack of acknowledgment and recognition by formal healthcare staff demotivated CHVs [36, 44, 46]. According to Lehmann & Sanders and Glenton et al., the relationship between CHVs and the formal health workers is dependent on how CHV programmes are introduced to the different stakeholders [7, 27]. In case they are introduced without proper engagement or involvement of the different stakeholders, then there are bound to experience some resistance [57]. For instance, a study by Woodgate (2007) showed that health professionals viewed CHVs as an extra workload, whereas others saw them as a threat to their authority. This in turn made healthcare workers have a negative attitude towards CHVs [57, 58]. According to Kok et al., negative attitudes between formal healthcare workers and CHVs resulted in communication challenges between the two groups, and adversely affected the community-health facility referral process [12]. There is therefore a need for all relevant stakeholders to be engaged when introducing CHV programmes at the primary healthcare level. During engagement, there is a need for proper briefing. Programmes should also have appropriate communication systems in place to ensure smooth transfer of information at all levels of the health system. It is important to note that the closer the collaboration between CHVs and formal healthcare workers, the better the working relationship [27].

Family support of the role played by a CHV was found to be critical for the success of any CHV programme. Disapproval of any CHV by the family led to CHV dropout [9, 36, 40]. This finding supports that by Kok et al., [12]. In their 2015 review, it was reported that husbands, mothers-in-law and grandmothers were the primary family decision-makers. As such CHVs, who were mostly married women, had to consult the relevant ‘family authority’ before making any decisions. This finding also concurs with the review by Lehmann & Sanders, and two other studies from Bangladesh, which found that for CHVs to be accepted in their communities, the roles played had to respond to the local, cultural and societal norms and customs [7, 50, 59]. This implies that to improve the CHVs performance, there is need to sensitize local community leaders including family heads on the role of CHVs to elicit their buy-in.

In the present review, support from supervisors and other formal health workers was reported to be an important source of motivation for CHVs working in urban informal settlements. The review by Glenton et al., reported similar findings in which CHV credibility in the community was strengthened by their connection to the formal health system [27]. Other authors have also demonstrated that lack of visible support from the health system weakened the CHVs’ credibility, thus negatively impacting on community trust [60].

CHV individual characteristics did not emerge strongly as a key theme in the reviewed papers, except for studies from India and Bangladesh [9, 36, 40]. It is unclear why this was the case. However, one potential reason could be that study authors did not deliberately inquire on the influence of such characteristics in the role of CHVs and the subsequent impact on performance. According to Kok et al., one of the CHVs’ characteristics with a positive influence on CHV performance was the CHVs’ prior experience with the health condition that they focused on. The age of a CHV had mixed effects on CHV performance [12]. It was also noted to be an important predictor by Sarma et al. although not always [50].

Personal safety at the community level was noted to be of concern to CHVs who lived and worked in dangerous neighbourhoods with high criminal activities such as prostitution, drug use and violence. Personal safety of CHVs was especially of concern when they had to work late into the evenings. This finding supports that of Glenton et al., which noted that safety was of concern amongst CHVs working in urban settings, as a result of poverty-related and social problems such as violence and drug use [27].

Specifically, in Kenyan urban and peri-urban informal settlements, the community expected ‘tangible support’ such as monetary aid from CHVs. As such, out of sympathy, some CHVs dipped into their own pockets to provide this support. This was to increase their acceptance in the community. Those CHVs who were not able to meet the households’ demand lost favour with the community thus causing them to dropout. To our knowledge, this finding has not been discussed elsewhere in the literature and should be interrogated in other LMICs.

## Limitations of the study and recommendations for future research

Only studies published in English were included in this review due to time and resource constraints. This resulted in exclusion of studies from some regions such as Latin America. This might have contributed to the low number of studies recorded. Nonetheless, the low numbers were indicative of a need for more in-depth qualitative studies, on factors that influence the performance of CHVs working in urban informal settlements.

Despite the above limitations, this review used an expert medical librarian in designing the search string. In addition, the review used three reviewers to standardize the data extraction process as well as the CASP, MMAST and CERQual approaches to assess for the quality and confidence of the review findings.

## Conclusion

This systematic synthesis of evidence highlights the programme design factors and broader contextual factors that CHV programmes being implemented in urban informal settlements should take into consideration to ensure success. These include adequate remuneration of CHVs; integrated and holistic training; supportive supervision; positive family support; appropriate health system linkages including adequate resources and supplies; personal safety issues; and community expectations such as the need for fiscal support to community households.

## Supplementary Information

**Additional file 1.** Database search strategy.

**Additional file 2.** Synonym of key words used in the search strategy.

**Additional file 3.** Excel database summary of all reviews.

## Data Availability

All data generated or analysed during this study are included in this published article [and its Additional files 1, 2 and 3].

## Abbreviations

CHV: Community health volunteer
CHW: Community health worker
LMIC: Low- and middle-income country
UHC: Universal health coverage
PHC: Primary healthcare
PEOS: Population, Exposure, Outcome, Study
EMBASE: Excerpta Medica database
CINAHL: The Cumulative Index of Nursing and Allied Health Literature
MO: Michael Ogutu
CD: Catherine Darker
KM: Kui Muraya
DM: David Mockler
PRISMA: Preferred Reporting Items for Systematic Reviews and Meta-analysis
CASP: Critical Appraisal Skills Program
CERQual: Confidence in the Evidence from Reviews of Qualitative Research
JBI: Joanna Briggs Institute
MMAST: Mixed Methods Assessment Tool
