# Supplementary material for: Factors influencing the performance of community health volunteers working within urban informal settlements in low- and middle-income countries: a qualitative meta-synthesis review
Source: Hum Resour Health. 2021 Nov 27;19:144. doi: 10.1186/s12960-021-00691-z (PMC8626887; doi:10.1186/s12960-021-00691-z)
Supplement: Supplementary file 1 — Additional file 1. Database search strategy. [file 12960_2021_691_MOESM1_ESM.pdf]

Community health volunteers

EMBASE

1. ('health care delivery'/exp OR 'community care'/exp OR 'community dwelling person'/exp OR 'community health nursing'/exp OR 'community medicine'/exp OR 'health auxiliary'/exp OR 'maternal health service'/exp OR 'maternal care'/exp) AND ('voluntary worker'/exp OR 'volunteer'/de OR 'community participation'/exp)
2. 'traditional birth attendant'/exp
3. (‘accredited social health activist*’ OR ‘ASHA worker*’ OR ‘barefoot doctor*’ OR ‘medical auxil?iar*’ OR monitrice* OR Linkworker* OR link-worker*):ti,ab
4. ((Community OR auxil?iary OR community-based OR village) NEAR/3 (‘health worker*’ OR ‘health care worker*’ OR ‘healthcare worker*’ OR ‘health aide*’ OR ‘health officer*’ OR practitioner* OR ‘health volunteer*’ OR nurs* OR midwi?e*)):ti,ab
5. ((lay OR voluntary OR volunteer OR volunteers) NEAR/3 (worker* OR attendant* OR aide* OR support* OR person* OR people OR helper* OR carer* OR caregiver* OR consultant* OR assistant* OR staff OR midwi?e* OR provider* OR 'care giver*' OR practitioner*)):ti,ab
6. ((untrained OR unlicensed OR non-professional* OR nonprofessional* OR informal OR non-formal OR ‘non formal’) NEAR/3 (worker* OR attendant* OR aide* OR support* OR person* OR people OR helper* OR carer* OR caregiver* OR consultant* OR assistant* OR staff OR midwi?e* OR provider* OR 'care giver*' OR practitioner*)):ti,ab
7. (trained NEAR/3 (volunteer* OR 'health worker*' OR mother*)):ti,ab
8. ((communit OR 'community based') NEAR/3 (volunteer* OR aide* OR support*)):ti,ab
9. ((birth OR childbirth OR labo?r) NEXT/1 (attendant* OR assistant*)):ti,ab
10. ((lay OR peer) NEXT/1 (volunteer* OR mentor* OR counsel* OR support* OR intervention*)):ti,ab
11. ('church based' NEAR/3 (intervention* OR program* OR counsel*)):ti,ab
12. (home NEXT/1 (care OR aide* OR nursing OR support OR intervention* OR treatment* OR visit*)):ti,ab
13. ((care OR aide* OR nursing OR nurse OR nurses OR support OR intervention* OR treatment* OR visit*) NEAR/3 (lay OR volunteer* OR voluntary)):ti,ab
14. ('expert patient*' OR 'health promoter*' OR 'health extension worker*' OR 'mentor mother*'):ti,ab
15. (‘community midwi?e*’ OR ‘lay birth attendant*’ OR ‘lay midwi?e’ OR ‘traditional midwi?e*’):ti,ab
16. #1 OR #2 OR #3 OR #4 OR #5 OR #6 OR #7 OR #8 OR #9 OR #10 OR #11 OR #12 OR #13 OR #14 OR #15
17. ('poverty'/exp OR 'lowest income group'/exp) AND ('urban population'/exp OR 'urban area'/exp OR 'urbanization'/exp)
18. 'urban slum'/exp
19. ((Informal OR irregular OR illegal OR squatter) NEAR/2 (shack* OR settlement* OR tenement*)):ti,ab
20. ((immigrant* OR migrant* OR refugee*) NEAR/2 (camp* OR settlement*)):ti,ab
21. (slum or slums or shanty or shanties or shantyhouse* or shanty-town* or barrio or barrios or favela* or favella* or ‘pueblo joven*’ OR kampung or tugurio* or ghetto or ghettos or bidon or bidons or bidonville*):ti,ab
22. (‘shack dweller*’ or shackdweller or ‘shack town*’ or ‘skid row’ or ‘shack settlement*’ or arrabal or asentamiento*or campamento* or cantegril* or comuna or comunas or

barriada* or ‘Poblacion callampa’ or tugurio or precario or chacarita or ‘tent cit*’ or ‘informal cit*’ or imijondolo*):ti,ab
23. (bustee* or gecekondu* or chawls* or basti or masseque* or ‘squatter camp*’ or ‘squatter cit*’ or ‘katchi abadi*’ or dharavi or kibera or ‘cite soleil’ or khayelitsha or ‘orangi town’):ti,ab
24. (‘kartonsko naselje’ or ‘habitat precaire’ or ‘habitat spontane’ or ‘quartier irregulier’ or ‘asentamiento irregular’ or ‘colonia popular’ or ‘villa miseria’ or ‘ciudad perdida’ or ‘edina achouaia’ or ‘mudal safi’ or ‘pelli gewal’):ti,ab
25. (lahbach or elendsviertel or brarek or foundouks or tanake or aashwa or truschobi or taudis or morro or loteamento or comunidade or ahata or katra or watta or jhopadpatti or umjondolo or mabanda or kijiji or barraca or conventillos):ti,ab
26. (‘cardboard cit*’ or ‘tenement district*’ or ‘tenement hous*’ or ‘rundown neighborhood*’ or ‘rundown neighbourhood*’ or ‘rundown settlement*’):ti,ab
27. #17 OR #18 OR #19 OR #20 OR #21 OR #22 OR #23 OR #24 OR #25 OR #26
28. ((('semi structured' OR semistructured OR unstructured OR informal OR 'in depth' OR indepth OR 'face to face' OR structured OR guide) NEAR/3 (interview* OR discussion* OR questionnaire*)):ti,ab) OR 'focus group*':ti,ab OR qualitative:ti,ab OR ethnograph*:ti,ab OR fieldwork:ti,ab OR 'field work':ti,ab OR 'key informant':ti,ab OR 'qualitative research'/de
29. interview*:ab,ti OR qualitative:ab,ti OR 'health care organization'/exp
30. #28 OR #29
31. #16 AND #27 AND #30

Medline(OVID)

1. exp Volunteers/ AND ("Community Health Workers"/ OR exp "Community Health Services"/ OR exp "Maternal Health Services"/ OR exp Health Services Accessibility/)
2. (accredited social health activist* OR ASHA worker* OR barefoot doctor* OR medical auxil?iar* OR monitrice* OR Linkworker* OR link-worker*).ti,ab.
3. ((Community OR auxil?iary OR community-based OR village) adj3 (health worker* OR health care worker* OR healthcare worker* OR health aide* OR health officer* OR practitioner* OR health volunteer* OR nurs* OR midwi?e*)).ti,ab.
4. ((lay OR voluntary OR volunteer OR volunteers) adj3 (worker* OR attendant* OR aide* OR support* OR person* OR people OR helper* OR carer* OR caregiver* OR consultant* OR assistant* OR staff OR midwi?e* OR provider* OR care giver* OR practitioner*)).ti,ab.
5. ((untrained OR unlicensed OR non-professional* OR nonprofessional* OR informal OR non-formal OR non formal) adj3 (worker* OR attendant* OR aide* OR support* OR person* OR people OR helper* OR carer* OR caregiver* OR consultant* OR assistant* OR staff OR midwi?e* OR provider* OR care giver* OR practitioner*)).ti,ab.
6. (trained adj3 (volunteer* OR health worker* OR mother*)).ti,ab.
7. ((communit OR community based) adj3 (volunteer* OR aide* OR support*)).ti,ab.
8. ((birth OR childbirth OR labo?r) adj1 (attendant* OR assistant*)).ti,ab.
9. ((lay OR peer) adj1 (volunteer* OR mentor* OR counsel* OR support* OR intervention*)).ti,ab.
10. (church based adj3 (intervention* OR program* OR counsel*)).ti,ab.
11. (home adj1 (care OR aide* OR nursing OR support OR intervention* OR treatment* OR visit*)).ti,ab.

12. ((care OR aide* OR nursing OR nurse OR nurses OR support OR intervention* OR treatment* OR visit*) ADJ3 (lay OR volunteer* OR voluntary)).ti,ab.
13. (expert patient* OR health promoter* OR health extension worker* OR mentor mother*).ti,ab.
14. (community midwi?e* OR lay birth attendant OR lay midwi?e OR traditional midwi?e).ti,ab.
15. or/1-14
16. exp poverty/ AND urban population/
17. ((Informal OR irregular OR illegal OR squatter) adj2 (shack* OR settlement* OR tenement*)).ti,ab.
18. ((immigrant* OR migrant* OR refugee*) adj2 (camp* OR settlement*)).ti,ab.
19. (slum or slums or shanty or shanties or shantyhouse* or shanty-town* or barrio or barrios or favela* or favella* or pueblo joven* OR kampung or tugurio* or ghetto or ghettos or bidon or bidons or bidonville*).ti,ab.
20. (shack dweller* or shackdweller or shack town* or skid row or shack settlement* or arrabal or asentamiento*or campamento* or cantegril* or comuna or comunas or barriada* or Poblacion callampa or tugurio or precario or chacarita or tent cit* or informal cit* or imijondolo*).ti,ab.
21. (bustee* or gecekondu* or chawls* or basti or masseque* or squatter camp* or squatter cit* or katchi abadi* or dharavi or kibera or cite soleil or khayelitsha or orangi town).ti,ab.
22. (kartonsko naselje or habitat precaire or habitat spontane or quartier irregulier or asentamiento irregular or colonia popular or villa miseria or ciudad perdida or edina achouaia or mudal safi or pelli gewal).ti,ab.
23. (lahbach or elendsviertel or brarek or foundouks or tanake or aashwa or truschobi or taudis or morro or loteamento or comunidade or ahata or katra or watta or jhopadpatti or umjondolo or mabanda or kijiji or barraca or conventillos).ti,ab.
24. (cardboard cit* or tenement district* or tenement hous* or rundown neighborhood* or rundown neighbourhood* or rundown settlement*).ti,ab.
25. or/16-24
26. and/15,25
27. ((("semi-structured" or semistructured or unstructured or informal or "in-depth" or indepth or "face-to-face" or structured or guide) adj3 (interview* or discussion* or questionnaire*))).ti,ab. or (focus group* or qualitative or ethnograph* or fieldwork or "field work" or "key informant").ti,ab. or interviews as topic/ or focus groups/ or narration/ or qualitative research/
28. 26 and 27

CINAHL

1. (MH "Community Health Workers") OR (MH "Community Health Services") OR (MH "Community Health Nursing") OR (MH "Volunteer Workers") OR (MH "Lay Midwives") OR (MH "Lay Midwifery")
2. TI (“accredited social health activist*” OR “ASHA worker*” OR “barefoot doctor*” OR “medical auxil?iar*” OR monitrice* OR Linkworker* OR link-worker*) OR AB (“accredited social health activist*” OR “ASHA worker*” OR “barefoot doctor*” OR “medical auxil?iar*” OR monitrice* OR Linkworker* OR link-worker*)
3. TI ((Community OR auxil?iary OR community-based OR village) N3 (“health worker*” OR “health care worker*” OR “healthcare worker*” OR “health aide*” OR “health officer*”

OR practitioner* OR “health volunteer*” OR nurs* OR midwi?e*)) OR AB ((Community OR auxil?iary OR community-based OR village) N3 (“health worker*” OR “health care worker*” OR “healthcare worker*” OR “health aide*” OR “health officer*” OR practitioner* OR “health volunteer*” OR nurs* OR midwi?e*))

4. TI ((lay OR voluntary OR volunteer OR volunteers) N3 (worker* OR attendant* OR aide* OR support* OR person* OR people OR helper* OR carer* OR caregiver* OR consultant* OR assistant* OR staff OR midwi?e* OR provider* OR “care giver*” OR practitioner*)) OR AB ((lay OR voluntary OR volunteer OR volunteers) N3 (worker* OR attendant* OR aide* OR support* OR person* OR people OR helper* OR carer* OR caregiver* OR consultant* OR assistant* OR staff OR midwi?e* OR provider* OR “care giver*” OR practitioner*))
5. TI ((untrained OR unlicensed OR non-professional* OR nonprofessional* OR informal OR non-formal OR “non formal”) N3 (worker* OR attendant* OR aide* OR support* OR person* OR people OR helper* OR carer* OR caregiver* OR consultant* OR assistant* OR staff OR midwi?e* OR provider* OR “care giver*” OR practitioner*)) OR AB ((untrained OR unlicensed OR non-professional* OR nonprofessional* OR informal OR non-formal OR “non formal”) N3 (worker* OR attendant* OR aide* OR support* OR person* OR people OR helper* OR carer* OR caregiver* OR consultant* OR assistant* OR staff OR midwi?e* OR provider* OR “care giver*” OR practitioner*))
6. TI (trained N3 (volunteer* OR “health worker*” OR mother*)) OR AB (trained N3 (volunteer* OR “health worker*” OR mother*))
7. TI ((communit OR “community based”) N3 (volunteer* OR aide* OR support*)) OR AB ((communit OR “community based”) N3 (volunteer* OR aide* OR support*))
8. TI ((birth OR childbirth OR labo?r) NEXT/1 (attendant* OR assistant*)) OR AB ((birth OR childbirth OR labo?r) NEXT/1 (attendant* OR assistant*))
9. TI ((lay OR peer) NEXT/1 (volunteer* OR mentor* OR counsel* OR support* OR intervention*)) OR AB ((lay OR peer) NEXT/1 (volunteer* OR mentor* OR counsel* OR support* OR intervention*))
10. TI (“church based” N3 (intervention* OR program* OR counsel*)) OR AB (“church based” N3 (intervention* OR program* OR counsel*))
11. TI (home NEXT/1 (care OR aide* OR nursing OR support OR intervention* OR treatment* OR visit*)) OR AB (home NEXT/1 (care OR aide* OR nursing OR support OR intervention* OR treatment* OR visit*))
12. TI ((care OR aide* OR nursing OR nurse OR nurses OR support OR intervention* OR treatment* OR visit*) N3 (lay OR volunteer* OR voluntary)) OR AB ((care OR aide* OR nursing OR nurse OR nurses OR support OR intervention* OR treatment* OR visit*) N3 (lay OR volunteer* OR voluntary))
13. TI (“expert patient*” OR “health promoter*” OR “health extension worker*” OR “mentor mother*”) OR AB (“expert patient*” OR “health promoter*” OR “health extension worker*” OR “mentor mother*”)
14. TI (“community midwi?e*” OR “lay birth attendant*” OR “lay midwi?e” OR “traditional midwi?e*”) OR AB (“community midwi?e*” OR “lay birth attendant*” OR “lay midwi?e” OR “traditional midwi?e*”)
15. S1 OR S2 OR S3 OR S4 OR S5 OR S6 OR S7 OR S8 OR S9 OR S10 OR S11 OR S12 OR S13 OR S14
16. ((MH "Poverty Areas") OR (MH "Poverty")) AND ((MH "Urban Areas") OR (MH "Urban Population") OR (MH "Urban Health") OR (MH "Urban Health Services"))

17. TI ((Informal OR irregular OR illegal OR squatter) N2 (shack* OR settlement* OR tenement*)) OR AB ((Informal OR irregular OR illegal OR squatter) N2 (shack* OR settlement* OR tenement*))
18. TI ((immigrant* OR migrant* OR refugee*) N2 (camp* OR settlement*)) OR AB ((immigrant* OR migrant* OR refugee*) N2 (camp* OR settlement*))
19. TI (slum or slums or shanty or shanties or shantyhouse* or shanty-town* or barrio or barrios or favela* or favella* or “pueblo joven*” OR kampung or tugurio* or ghetto or ghettos or bidon or bidons or bidonville*) OR AB (slum or slums or shanty or shanties or shantyhouse* or shanty-town* or barrio or barrios or favela* or favella* or “pueblo joven*” OR kampung or tugurio* or ghetto or ghettos or bidon or bidons or bidonville*)
20. TI (“shack dweller*” or shackdweller or “shack town*” or “skid row” or “shack settlement*” or arrabal or asentamiento*or campamento* or cantegril* or comuna or comunas or barriada* or “Poblacion callampa” or tugurio or precario or chacarita or “tent cit*” or “informal cit*” or imijondolo*) OR AB (“shack dweller*” or shackdweller or “shack town*” or “skid row” or “shack settlement*” or arrabal or asentamiento*or campamento* or cantegril* or comuna or comunas or barriada* or “Poblacion callampa” or tugurio or precario or chacarita or “tent cit*” or “informal cit*” or imijondolo*)
21. TI (bustee* or gecekondu* or chawls* or basti or masseque* or “squatter camp*” or “squatter cit*” or “katchi abadi*” or dharavi or kibera or “cite soleil” or khayelitsha or “orangi town”) OR AB (bustee* or gecekondu* or chawls* or basti or masseque* or “squatter camp*” or “squatter cit*” or “katchi abadi*” or dharavi or kibera or “cite soleil” or khayelitsha or “orangi town”)
22. TI (“kartonsko naselje” or “habitat precaire” or “habitat spontane” or “quartier irregulier” or “asentamiento irregular” or “colonia popular” or “villa miseria” or “ciudad perdida” or “edina achouaia” or “mudal safi” or “pelli gewal”) OR AB (“kartonsko naselje” or “habitat precaire” or “habitat spontane” or “quartier irregulier” or “asentamiento irregular” or “colonia popular” or “villa miseria” or “ciudad perdida” or “edina achouaia” or “mudal safi” or “pelli gewal”)
23. TI (lahbach or elendsviertel or brarek or foundouks or tanake or aashwa or truschobi or taudis or morro or loteamento or comunidade or ahata or katra or watta or jhopadpatti or umjondolo or mabanda or kijiji or barraca or conventillos) OR AB (lahbach or elendsviertel or brarek or foundouks or tanake or aashwa or truschobi or taudis or morro or loteamento or comunidade or ahata or katra or watta or jhopadpatti or umjondolo or mabanda or kijiji or barraca or conventillos)
24. TI (“cardboard cit*” or “tenement district*” or “tenement hous*” or “rundown neighborhood*” or “rundown neighbourhood*” or “rundown settlement*”) OR AB (“cardboard cit*” or “tenement district*” or “tenement hous*” or “rundown neighborhood*” or “rundown neighbourhood*” or “rundown settlement*”)
25. S16 OR S17 OR S18 OR S19 OR S20 OR S21 OR S22 OR S23 OR S24
26. (MH "cluster sample+") or TX life experiences or TX human science or TX discourse* analysis or TX narrative analysis or TX lived experience* or TX field research or TX field studies or TX field study or TX giorgi* or TX husserl* or TX merleau ponty* or TX van kaam* or TX van manen* or TX spiegelberg* or TX colaizzi* or TX heidegger* or TX participant observ* or TX data saturat* or TX semiotics or TX heuristic or TX hermeneutic* or TX etic or TX emic or TX focus group* or TX purpos* sampl* or TX constant comparison or TX constant comparative or TX grounded research or TX grounded studies or TX grounded study or TX grounded theor* or TX phenomenol* or TX ethnon* or TX qualitative or (MH "ethnological research") or (MH "ethnography") or

(MH "phenomenology") or (MH "focus groups") or (MH "discourse analysis") or (MH "theoretical sample") or (MH "field studies") or (MH "constant comparative method") or (MH "thematic analysis") or (MH "content analysis") or (MH "observational methods+") or (MH "purposive sample") or (MH "qualitative validity+") or (MH "grounded theory") or (MH “action research”) or (MH “naturalistic inquiry”) or (MH "ethnonursing research") or (MH "phenomenological research") or (MH "ethnographic research") or (MH "qualitative studies") or (MH "Interviews+") or (MH "Narratives") or (MH "Videorecording+") or (MH "Audiorecording") or (MH "Historical Records")

27. S15 AND S25 AND S26

Web of Science

1. TI =(“accredited social health activist*” OR “ASHA worker*” OR “barefoot doctor*” OR “medical auxil?iar*” OR monitrice* OR Linkworker* OR link-worker*) OR AB =(“accredited social health activist*” OR “ASHA worker*” OR “barefoot doctor*” OR “medical auxil?iar*” OR monitrice* OR Linkworker* OR link-worker*)
2. Ti =((Community OR auxil?iary OR community-based OR village) NEAR/3 (“health worker*” OR “health care worker*” OR “healthcare worker*” OR “health aide*” OR “health officer*” OR practitioner* OR “health volunteer*” OR nurs* OR midwi?e*)) OR AB =((Community OR auxil?iary OR community-based OR village) NEAR/3 (“health worker*” OR “health care worker*” OR “healthcare worker*” OR “health aide*” OR “health officer*” OR practitioner* OR “health volunteer*” OR nurs* OR midwi?e*))
3. TI =((lay OR voluntary OR volunteer OR volunteers) NEAR/3 (worker* OR attendant* OR aide* OR support* OR person* OR people OR helper* OR carer* OR caregiver* OR consultant* OR assistant* OR staff OR midwi?e* OR provider* OR “care giver*” OR practitioner*)) OR AB =((lay OR voluntary OR volunteer OR volunteers) NEAR/3 (worker* OR attendant* OR aide* OR support* OR person* OR people OR helper* OR carer* OR caregiver* OR consultant* OR assistant* OR staff OR midwi?e* OR provider* OR “care giver*” OR practitioner*))
4. TI =((untrained OR unlicensed OR non-professional* OR nonprofessional* OR informal OR non-formal OR “non formal”) NEAR/3 (worker* OR attendant* OR aide* OR support* OR person* OR people OR helper* OR carer* OR caregiver* OR consultant* OR assistant* OR staff OR midwi?e* OR provider* OR “care giver*” OR practitioner*)) OR AB =((untrained OR unlicensed OR non-professional* OR nonprofessional* OR informal OR non-formal OR “non formal”) NEAR/3 (worker* OR attendant* OR aide* OR support* OR person* OR people OR helper* OR carer* OR caregiver* OR consultant* OR assistant* OR staff OR midwi?e* OR provider* OR “care giver*” OR practitioner*))
5. TI =(trained NEAR/3 (volunteer* OR “health worker*” OR mother*)) OR AB =(trained NEAR/3 (volunteer* OR “health worker*” OR mother*))
6. TI =((communit OR “community based”) NEAR/3 (volunteer* OR aide* OR support*)) OR AB =((communit OR “community based”) NEAR/3 (volunteer* OR aide* OR support*))
7. TI =((birth OR childbirth OR labo?r) NEAR/1 (attendant* OR assistant*)) OR AB =((birth OR childbirth OR labo?r) NEAR/1 (attendant* OR assistant*))
8. TI =((lay OR peer) NEAR/1 (volunteer* OR mentor* OR counsel* OR support* OR intervention*)) OR AB =((lay OR peer) NEAR/1 (volunteer* OR mentor* OR counsel* OR support* OR intervention*))
9. TI =(“church based” NEAR/3 (intervention* OR program* OR counsel*)) OR AB =(“church based” NEAR/3 (intervention* OR program* OR counsel*))

10. TI =(home NEAR/1 (care OR aide* OR nursing OR support OR intervention* OR treatment* OR visit*)) OR AB =(home NEAR/1 (care OR aide* OR nursing OR support OR intervention* OR treatment* OR visit*))
11. TI =((care OR aide* OR nursing OR nurse OR nurses OR support OR intervention* OR treatment* OR visit*) NEAR/3 (lay OR volunteer* OR voluntary)) OR AB =((care OR aide* OR nursing OR nurse OR nurses OR support OR intervention* OR treatment* OR visit*) NEAR/3 (lay OR volunteer* OR voluntary))
12. TI =("expert patient*" OR "health promoter*" OR "health extension worker*" OR "mentor mother*" OR "community midwi?e*" OR "lay birth attendant*" OR "lay midwi?e" OR "traditional midwi?e*") OR AB=("expert patient*" OR "health promoter*" OR "health extension worker*" OR "mentor mother*" OR "community midwi?e*" OR "lay birth attendant*" OR "lay midwi?e" OR "traditional midwi?e*")
13. #1 OR #2 OR #3 OR #4 OR #5 OR #6 OR #7 OR #8 OR #9 OR #10 OR #11 OR #12
14. TI = (((Informal OR irregular OR illegal OR squatter) NEAR/2 (shack* OR settlement* OR tenement*)) OR ((immigrant* OR migrant* OR refugee*) NEAR/2 (camp* OR settlement*)) OR (slum or slums or shanty or shanties or shantyhouse* or shanty-town* or barrio or barrios or favela* or favella* or "pueblo joven*" OR kampung or tugurio* or ghetto or ghettos or bidon or bidons or bidonville*) OR ("shack dweller*" or shackdweller or "shack town*" or "skid row" or "shack settlement*" or arrabal or asentamiento*or campamento* or cantegril* or comuna or comunas or barriada* or "Poblacion callampa" or tugurio or precario or chacarita or "tent cit*" or "informal cit*" or imijondolo*) OR (bustee* or gecekondu* or chawls* or basti or masseque* or "squatter camp*" or "squatter cit*" or "katchi abadi*" or dharavi or kibera or "cite soleil" or khayelitsha or "orangi town") OR ("kartonsko naselje" or "habitat precaire" or "habitat spontane" or "quartier irregulier" or "asentamiento irregular" or "colonia popular" or "villa miseria" or "ciudad perdida" or "edina achouaia" or "mudal safi" or "pelli gewal") OR (lahbach or elendsviertel or brarek or foundouks or tanake or aashwa or truschobi or taudis or morro or loteamento or comunidade or ahata or katra or watta or jhopadpatti or umjondolo or mabanda or kijiji or barraca or conventillos) OR ("cardboard cit*" or "tenement district*" or "tenement hous*" or "rundown neighborhood*" or "rundown neighbourhood*" or "rundown settlement*"))
15. AB = (((Informal OR irregular OR illegal OR squatter) NEAR/2 (shack* OR settlement* OR tenement*)) OR ((immigrant* OR migrant* OR refugee*) NEAR/2 (camp* OR settlement*)) OR (slum or slums or shanty or shanties or shantyhouse* or shanty-town* or barrio or barrios or favela* or favella* or "pueblo joven*" OR kampung or tugurio* or ghetto or ghettos or bidon or bidons or bidonville*) OR ("shack dweller*" or shackdweller or "shack town*" or "skid row" or "shack settlement*" or arrabal or asentamiento*or campamento* or cantegril* or comuna or comunas or barriada* or "Poblacion callampa" or tugurio or precario or chacarita or "tent cit*" or "informal cit*" or imijondolo*) OR (bustee* or gecekondu* or chawls* or basti or masseque* or "squatter camp*" or "squatter cit*" or "katchi abadi*" or dharavi or kibera or "cite soleil" or khayelitsha or "orangi town") OR ("kartonsko naselje" or "habitat precaire" or "habitat spontane" or "quartier irregulier" or "asentamiento irregular" or "colonia popular" or "villa miseria" or "ciudad perdida" or "edina achouaia" or "mudal safi" or "pelli gewal") OR (lahbach or elendsviertel or brarek or foundouks or tanake or aashwa or truschobi or taudis or morro or loteamento or comunidade or ahata or katra or watta or jhopadpatti or umjondolo or mabanda or kijiji or barraca or conventillos) OR

(“cardboard cit*” or “tenement district*” or “tenement hous*” or “rundown neighborhood*” or “rundown neighbourhood*” or “rundown settlement*”))

16. #14 OR #15
17. TI =(qualitative OR ethnol* OR ethnog* OR ethnonurs* OR emic OR etic OR leininger OR noblit OR "field note*" OR "field record*" OR fieldnote* OR "field stud*" or "participant observ*" OR "participant observation*" OR hermaneutic* OR phenomenolog* OR "lived experience*" OR heidegger* OR husserl* OR "merleau-pont*" OR colaizzi OR giorgi OR ricoeur OR spiegelberg OR "van kaam" OR "van manen" OR "grounded theory" OR "constant compar*" OR "theoretical sampl*" OR glaser AND strauss OR "content analy*" OR "thematic analy*" OR narrative* OR "unstructured categor*" OR "structured categor*" OR "unstructured interview*" OR "semi-structured interview*" OR "maximum variation*" OR snowball OR audio* OR tape* OR video* OR metasynthes* OR "meta-synthes*" OR metasummar* OR "meta-summar*" OR metastud* OR "meta-stud*" OR "meta-ethnograph*" OR metaethnog* OR "meta-narrative*" OR metanarrat* OR " meta-interpretation*" OR metainterpret* OR "qualitative meta-analy*" OR "qualitative metaanaly*" OR "qualitative metanaly*" OR "purposive sampl*" OR "action research" OR "focus group*" or photovoice or "photo voice" or "mixed method*") OR AB =(qualitative OR ethnol* OR ethnog* OR ethnonurs* OR emic OR etic OR leininger OR noblit OR "field note*" OR "field record*" OR fieldnote* OR "field stud*" or "participant observ*" OR "participant observation*" OR hermaneutic* OR phenomenolog* OR "lived experience*" OR heidegger* OR husserl* OR "merleau-pont*" OR colaizzi OR giorgi OR ricoeur OR spiegelberg OR "van kaam" OR "van manen" OR "grounded theory" OR "constant compar*" OR "theoretical sampl*" OR glaser AND strauss OR "content analy*" OR "thematic analy*" OR narrative* OR "unstructured categor*" OR "structured categor*" OR "unstructured interview*" OR "semi-structured interview*" OR "maximum variation*" OR snowball OR audio* OR tape* OR video* OR metasynthes* OR "meta-synthes*" OR metasummar* OR "meta-summar*" OR metastud* OR "meta-stud*" OR "meta-ethnograph*" OR metaethnog* OR "meta-narrative*" OR metanarrat* OR " meta-interpretation*" OR metainterpret* OR "qualitative meta-analy*" OR "qualitative metaanaly*" OR "qualitative metanaly*" OR "purposive sampl*" OR "action research" OR "focus group*" or photovoice or "photo voice" or "mixed method*")
18. #16 AND #13 AND #17
