# Supplementary material for: Factors influencing the performance of community health volunteers working within urban informal settlements in low- and middle-income countries: a qualitative meta-synthesis review
Source: Hum Resour Health. 2021 Nov 27;19:144. doi: 10.1186/s12960-021-00691-z (PMC8626887; doi:10.1186/s12960-021-00691-z)
Supplement: Supplementary file 2 — Additional file 2. Synonym of key words used in the search strategy. [file 12960_2021_691_MOESM2_ESM.pdf]

Table of synonyms of key words used in developing the search strategy

Population 1	Population 2	Population 3	Population 4	Exposure	Outcome 1	Outcome 2	Outcome 3	Study
CHV search terms as outlined in Table 6	Urban, City, Cities	Informal/ irregular/ illegal slum, settlement/shack/squatter/ tenement/shanty	Low-and Middle-income countries, LMIC, Low Income Countries, Low Income Country, Middle Income Countries, Middle Income Country, Africa, Asia, developing country, developing countries	CHV programme, CHV programmes	(Intervention, Program characteristics): performance appraisal, personnel selection, personnel recruitment, personnel turnover, staff development, workload, motivation, renumeration, incentives, disincentives, job satisfaction, job performance, retention, supervision, task shifting, quality assurance, continuing education, management quality circles, monitoring and evaluation, medical information system, medical information systems, mobile health, mhealth, ehealth, m-health, e-health, community participation, ownership, empowerment, gender, accountability, village health committees, village health committee, decentralization, decentralisation	(Health system factors): effectiveness, efficiency, equity, health care utilization, patient compliance, patient attitude, patient attitudes, health care quality, patient satisfaction, cost effectiveness analysis, cost benefit analysis	(Broad contextual factors): social, culture, norms, values, practices, beliefs, gender roles, legislation, politics, human resource policy	Qualitative, semi structured, unstructured, informal, in depth, face to face, focus group, key informant, interview, ethnography, narration
